# ACE2: the molecular doorway to SARS-CoV-2

**DOI:** 10.1186/s13578-020-00519-8

**Published:** 2020-12-30

**Authors:** Miriam Marlene Medina-Enríquez, Sandra Lopez-León, José Alberto Carlos-Escalante, Zuleika Aponte-Torres, Angelica Cuapio, Talia Wegman-Ostrosky

**Affiliations:** 1 Facultad de Farmacia, Universidad de Barcelona, Barcelona, Spain; 2 Global Drug Development, Novartis Pharmaceuticals Corporation, East Hanover, NJ USA; 3 PECEM (MD/PhD), Facultad de Medicina, Universidad Nacional Autónoma de México, Mexico City, Mexico; 4 MS Epidemiology Consultant, Santiago de Chile, Chile; 5 Center of Infectious Medicine, Karolinska Institutet, Stockholm, Sweden; 6 Department of Basic Research, Instituto Nacional de Cancerología, 22 San Fernando Avenue, Belisario Domínguez Sección XVI, 14080 Mexico City, Mexico

**Keywords:** ACE2, SARS-CoV-2, COVID-19, Coronavirus

## Abstract

The angiotensin-converting enzyme 2 (ACE2) is the host functional receptor for the new virus SARS-CoV-2 causing Coronavirus Disease 2019. ACE2 is expressed in 72 different cell types. Some factors that can affect the expression of the ACE2 are: sex, environment, comorbidities, medications (e.g. anti-hypertensives) and its interaction with other genes of the renin-angiotensin system and other pathways. Different factors can affect the risk of infection of SARS-CoV-2 and determine the severity of the symptoms. The ACE2 enzyme is a negative regulator of RAS expressed in various organ systems. It is with immunity, inflammation, increased coagulopathy, and cardiovascular disease. In this review, we describe the genetic and molecular functions of the ACE2 receptor and its relation with the physiological and pathological conditions to better understand how this receptor is involved in the pathogenesis of COVID-19. In addition, it reviews the different comorbidities that interact with SARS-CoV-2 in which also ACE2 plays an important role. It also describes the different factors that interact with the virus that have an influence in the expression and functional activities of the receptor. The goal is to provide the reader with an understanding of the complexity and importance of this receptor.

## Introduction

The angiotensin-converting enzyme 2 (ACE2) is a protein that has different roles such as catalytic, transporter of amino acids or viral receptor. It has an essential role in different systems, from cardiovascular regulation to viral infection.

The ACE attaches to cell membranes and works as the host functional receptor for the new virus SARS-CoV-2 which causes Coronavirus Disease 2019 (COVID-19). COVID-19 affects mainly the respiratory system; however, it can also affect different systems in the body. COVID-19 can be serious, it can produce multiple organ dysfunction syndrome (MODS) or death.

Coronaviruses (CoVs) are a family of RNA viruses. To date, 7 CoVs have been identified affecting humans 229E, OC43, NL63, HKU1, SARS-CoV, MERS-CoV and the new SARS-CoV-2. According to Zhou et al. [1] the genome of the new coronavirus, SARS-CoV-2 shares about 79% similitude with SARS-CoV and 96% identical at the whole-genome level to a bat CoV RaTG13 isolated from *Rhinolophus affinis*. Several studies suggest that the human coronavirus has a zoonotic origin [2, 3], and the Malayan pangolin (*Manis javanica*) is the potential natural reservoir or intermediate host of SARS-CoV-2 [3, 4].

The SARS-CoV and the SARS-CoV-2 enter the human cell through the ACE2 receptor. These two viruses have a surface anchored Spike (S) glycoprotein with surfaces receptor binding domains (RBD) [5, 6]. These two structures are critical for the entrance of the virus into the human cell. When the genetic material of the virus enters the cell, the membrane of the virus fusions with the host membrane cells [7, 8].

SARS-CoV-2 has a significantly higher ACE2 binding affinity [6]. According to Shang et al. [9], the 3-dimensional structure of the SARS-CoV-2 binding site has a more compact conformation, improved binding stability, and potentially enhances the ACE2 receptor binding affinity. Sequence-based prediction studies suggest a more efficient cleavage site inserted at the boundary of the S1/S2 subunits of the spike S protein (a host proprotein convertase, furin). This polybasic furin-type cleavage site is unique, can enhance the virus ability to internalize into cells [10]. Furthermore, studies through surface plasmon resonance have proven that the ACE2 binds to the ectodomain of the SARS-CoV-2 spike glycoprotein with about 10- to 20-fold higher affinity than the S protein of the previous SARS-CoV [11]. These different characteristics may explain the higher SARS-CoV-2 infectivity.

ACE2 is part of the Renin Angiotensin System (RAS), the main network responsible for the regulation of systemic arterial pressure. Besides its well-known systemic regulation of the circulatory homeostasis, the RAS also has a local or paracrine function. RAS is a complex system that is involved in multiple biological processes. The functions of the system are broad, some of the functions are antagonistic, which include inflammation, angiogenesis, cell proliferation, memory, sodium and water reabsorption, thrombosis and plaque rupture [12, 13]. Understanding the regulation of RAS, especially ACE2 in the context of COVID-19 could help understand the physiopathology of the disease and lead to the development of preventive and therapeutic drugs that help fight the pandemic disease.

The aim of this review is to describe the genetic and molecular functions of the ACE2 receptor in physiological and in pathological conditions to understand how this receptor is involved in the pathogenesis of COVID-19.

## *ACE2* gene

**Fig. 1:**
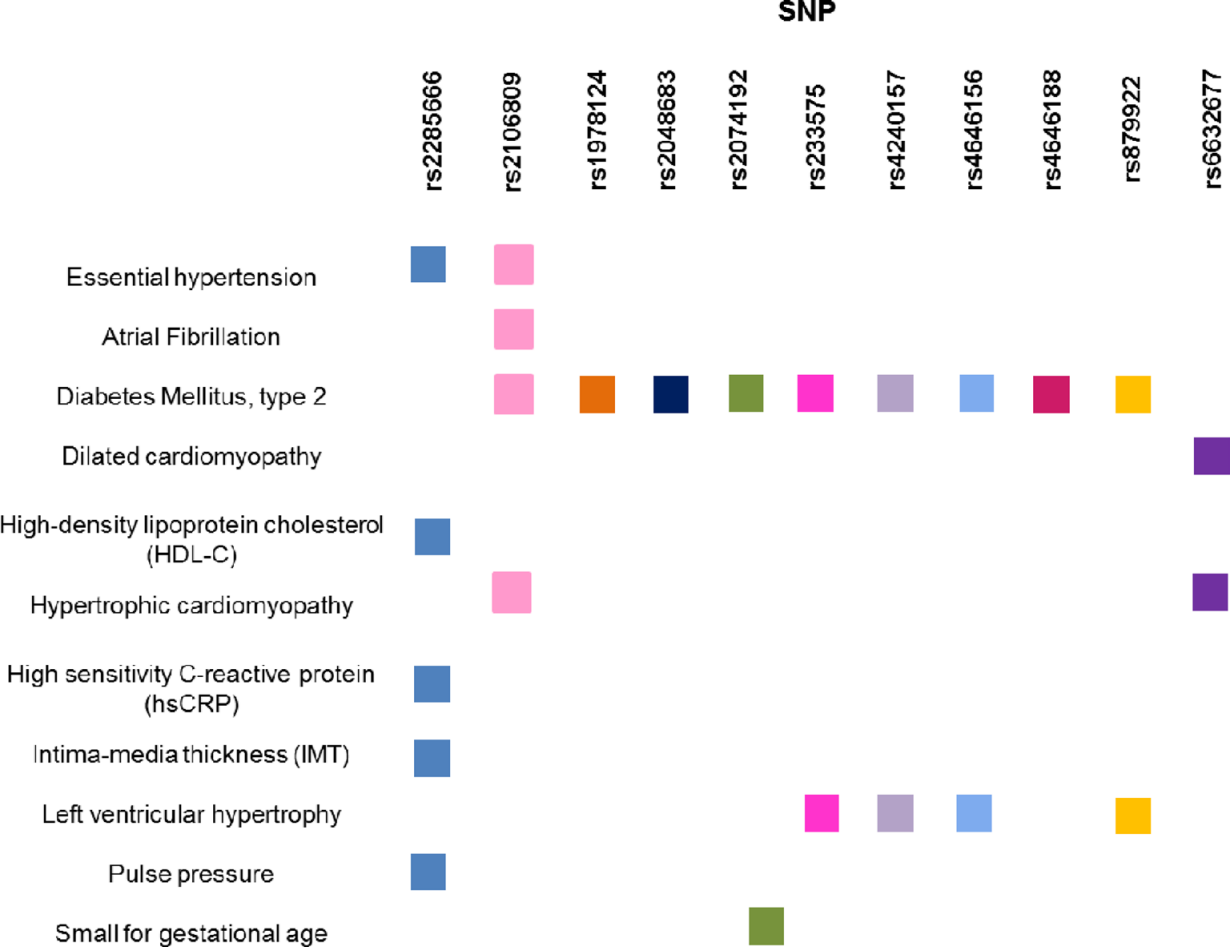
ACE2 SNPs associated diseases reported in meta-analyses. 11 genetic variants are represented by different colors and have been associated with different diseases; rs2285666 and rs2106809 are the most frequent genetic variants, rs2285666 is correlated with essential hypertension, HDL-C, hsCRP, IMT and pulse pressure and rs2106809 is linked to diabetes mellitus (type 2), atrial fibrillation and hypertrophic cardiomyopathy. This figure was created using Server Medical Art templates, which are licensed under a Creative Commons Attribution 3.0 Unported License; https://smart.servier.com

The *ACE2* gene codes for the angiotensin-converting enzyme 2 (ACE2) protein. It is located on chromosome Xp22 and contains 20 introns and 18 exons. It spans 40 kb of genomic DNA. *ACE2* could have evolved from *ACE* given that these two genes exhibit a 42% sequence homology. *ACE2* is a polymorphic gene with genetic variants that have been associated with several diseases including hypertension, atrial fibrillation, diabetes mellitus, dilated cardiomyopathy, HDL, hypertrophic cardiomyopathy, high sensitivity C-reactive protein, intima media thickness, left ventricular hypertrophy, pulse pressure and small for gestational age birth (Fig. 1, Additional file 1) [14]. It is interesting to note that all of these outcomes are either predisposing factors to develop severe COVID-19 disease, or are part of the symptomatology of the disease. In Fig. 1 we can see all the polymorphisms of the ACE2 that have been associated to different diseases which have been studied in meta-analyses.

Given that the *ACE2* gene is located in the X chromosome, some studies focused on assessing whether these associations are only present in males or females. For example, in a large cross-sectional study of the MONICA Augsburg echocardiographic substudy [15], four *ACE2* SNPs (rs4646156, rs879922, rs4240157 and rs233575) were significantly associated with LVH in men, but not in women. Another study on cardiac structure and function found in women that the *ACE2* rs1978124 A allele was significantly associated with an increased Left ventricular V mass. Given that there is a higher risk for men to develop severe forms of COVID-19, it would be relevant to stratify further analyses related to ACE2 and COVID-19 on gender.

So far, no *ACE2* genetic variant has been associated to increased risk of SARS-CoV-2 infection or to the severe clinical presentation of COVID-19 [16, 17]. An Italian study comparing 131 COVID-19 and 258 controls without the disease found that higher *ACE2* variability was present in the control group [18] suggesting that variants in *ACE2* could explain some differences in the susceptibility among different individuals. Several variants affecting the SARS-CoV-2 binding region of *ACE2* has been proposed (e.g. rs73635825, rs4646116 and rs766996587) as candidate variants to be studied in relation to COVID-19 [19] [preprint: not peer-reviewed], these variants still need to be assessed in large samples.

Further research is ongoing to determine if having certain genetic variants could increase the risk of developing certain symptoms of COVID-19. There is hope that further knowledge can guide on the prevention, diagnosis and treatment of this disease.

## ACE2 protein: the receptor and the soluble form

The human ACE2 protein is a zinc metallopeptidase, an ectoenzyme (family of dipeptidyl carboxydipeptidase), which contains 805 amino acids. This protein is a type I transmembrane glycoprotein and its expression is ubiquitous with a single extracellular catalytic domain that predominantly localizes at the plasma membrane [20, 21].

There are two functional forms of the ACE2 protein. The first form is the full-length ACE2 protein, which contains a structural transmembrane domain and spikes its extracellular domain to the plasma membrane. The second form is the soluble form, which lacks the membrane anchor and circulates in small amounts in the blood. ACE2 has also been shown to regulate cardiovascular functions in brain regions [22]. The soluble form represents the circulating ACE2 blood vessels. ACE2 plays a major role in balancing the levels of Angiotensin II (AngII) and Angiotensin-(1–7) (Ang (1–7) [23].

The role of the ACE2 soluble form (sACE2) in the pathogenesis of COVID-19 has not been completely elucidated. Age and gender differences in the soluble form of ACE2 (sACE2) among COVID-19 patients, show that adults and men exhibit higher plasma concentrations of sACE2 relative to children and women, respectively [24]. Since severe COVID-19 is more common in adults than in children and in men than women [25], it has been proposed that high sACE2 levels, reflecting high membrane-bound ACE2 levels, might lead to an increased susceptibility to SARS-CoV-2 infection [24]. However, this has not been proven conclusively. In contrast, a growing body of evidence suggests the protective role of sACE [26, 27] by its function as decoy ligand to sequester SARS-CoV-2 away from the membrane receptor ACE2 which internalize docked viruses via membrane-associated enzyme dynamics that determines SARS-CoV-2 tissue tropism [28]. In this line, a genetically modified soluble form of ACE2, called hrsACE2, designed to minimize lung injury and multiple organ dysfunction, competes for membrane-bound ACE2 and thus decreases the cell entry of SARS-CoV-2 into the target cells. Such hrsACE2 reduces viral growth of SARS-CoV-2 by a factor of 1000–5000 in cell culture, engineered human blood vessels and kidney organoids [29].

## ACE2 expression

ACE2 is widely expressed in many different cells of the body. In 2002, Harmer et al. [30] studied the expression of ACE2 and found that the mRNA is expressed in 72 different tissues obtained from three human donors. It was observed to be highly expressed in endocrine tissues, gastrointestinal tract (e.g. ileum, liver and gallbladder), cardiovascular tissues, kidney and urinary bladder, testes and muscle tissues. It was observed that central nervous system and lymphoid tissues express relatively low ACE2 levels. They found that that the receptor it is not expressed in red blood cells. In the lung, high mRNA ACE2 expression was detected in the parenchyma and in primary and tertiary bronchi. Relevant for the transmission and respiratory manifestations of SARS-CoV-2, ACE-2 positive cells were observed in oral, nasal, and nasopharynx epithelia, and in type I and type II alveolar epithelial cells (AT1 and AT2 cells).

A bioinformatic analysis by Wang et al. [31] reported that the small intestine, testis, kidney, heart muscle, colon and thyroid gland were the tissues expressing the largest quantities of ACE2 mRNA across 3 different transcriptome databases. Surprisingly, lung expression of ACE2 was rather low. Other studies have used single-cell RNA-seq (scRNA-seq) to study ACE2 expression. This technique identifies different transcriptomic profiles across several cell types and allows for a more precise quantification of cells expressing a gene of interest. In these studies, the proportion of AT2 cells expressing *ACE2* mRNA is around 1% [32, 33] For comparison, 30% of ileal epithelial cells expressed ACE2, 7.5% of myocardial cells, 4% of kidney proximal tubule cells and 2,4% of bladder urothelial cells [34].

Whether the fraction of AT2 cells expressing *ACE2* is enough for SARS-CoV-2 to establish an infection or other entry factors may play a role is still unknown. Nonetheless, it should be noted that scRNA-seq may underestimate the percentage of cells expressing ACE2 [33]. Additionally, ACE2 has been observed to be upregulated by interferons, typically elevated in humans during airway infections [33]. Therefore, it was suggested that the interferons secreted upon initial infection could potentiate SARS-CoV-2 further dissemination. However, Onabajo et al. [35] demonstrated that only a truncated form of ACE2 (termed dACE2) is induced by interferons and this isoform did not increase SARS-CoV-2 infection.

Some factors that can affect the expression of the ACE2 are: sex, genetic variants in the genes of the RAS system and other factors like the use of some antihypertensive drugs. All of these factors can affect the risk of infection of SARS-CoV-2 and determine the severity of the symptoms.

Sex-specific differences in the expression of the RAS components have been uncovered in mice. The female mice exhibit predominance in ACE2/Ang(1–7)/MasR in comparison with male mice [36]. The loss of the 17β-estradiol in postmenopause is associated with an increased cardiovascular risk in women, this is probably due to a change in the balance of the ACE2/Ang(1–7)/MasR axis and the ACE/AngII/AT1R axis [37]. Furthermore, scRNA-seq studies on COVID-19 have shown an association of an upregulation of ACE2 and related proteases expression in airway epithelial and AT2 cells that increase with age and mainly in men [38] [preprint: not peer-reviewed]. This can be an explanation for the increased COVID-19 mortality seen in aging males. Some evidence shows that women have a lower rate of COVID-19 disease severity but further clinical trials are needed. Two clinical trials have been initiated to examine whether short-term treatment of male COVID-19 positive patients with an estrogen patch (NCT04359329) or progesterone (NCT04365127) [39].

After adjusting for sex and the presence of asthma, it was noticed that the ACE2 gene expression in nasal epithelium is age dependent, with younger individuals expressing this gene less than adults. This finding may explain the reduced susceptibility to a SARS-CoV-2 infection observed in children and their milder clinical course [40]. It is unknown whether the same occurs in bronchial epithelium. A study by Schouten et al. found no differences in the activity of ACE2 measured in bronchoalveolar lavage fluid [41]. However, this might not be an indicator of receptor expression nor of ACE2 gene expression in bronchial epithelium.

ACE2 is expressed on type I and II alveolar epithelial cells in a normal human lung and the binding with SARS-CoV-2 provokes an elevated expression of this protein. Men had an elevated expression of ACE2 in their alveolar cells compared to women, while Asian people present in their alveolar cell a higher expression of ACE2 than white or black people [42]. Chen et al. [43] found by using the GTEx data, a higher ACE2 expression in Asian females, an age-dependent decrease in all ethnic groups. In addition, the group demonstrated that the expression of ACE2 is reduced in diabetic patients (type II) and with inflammatory cytokine treatment and upregulated by estrogen and androgen (both decrease with age).

## Physiological functions

**Fig. 2:**
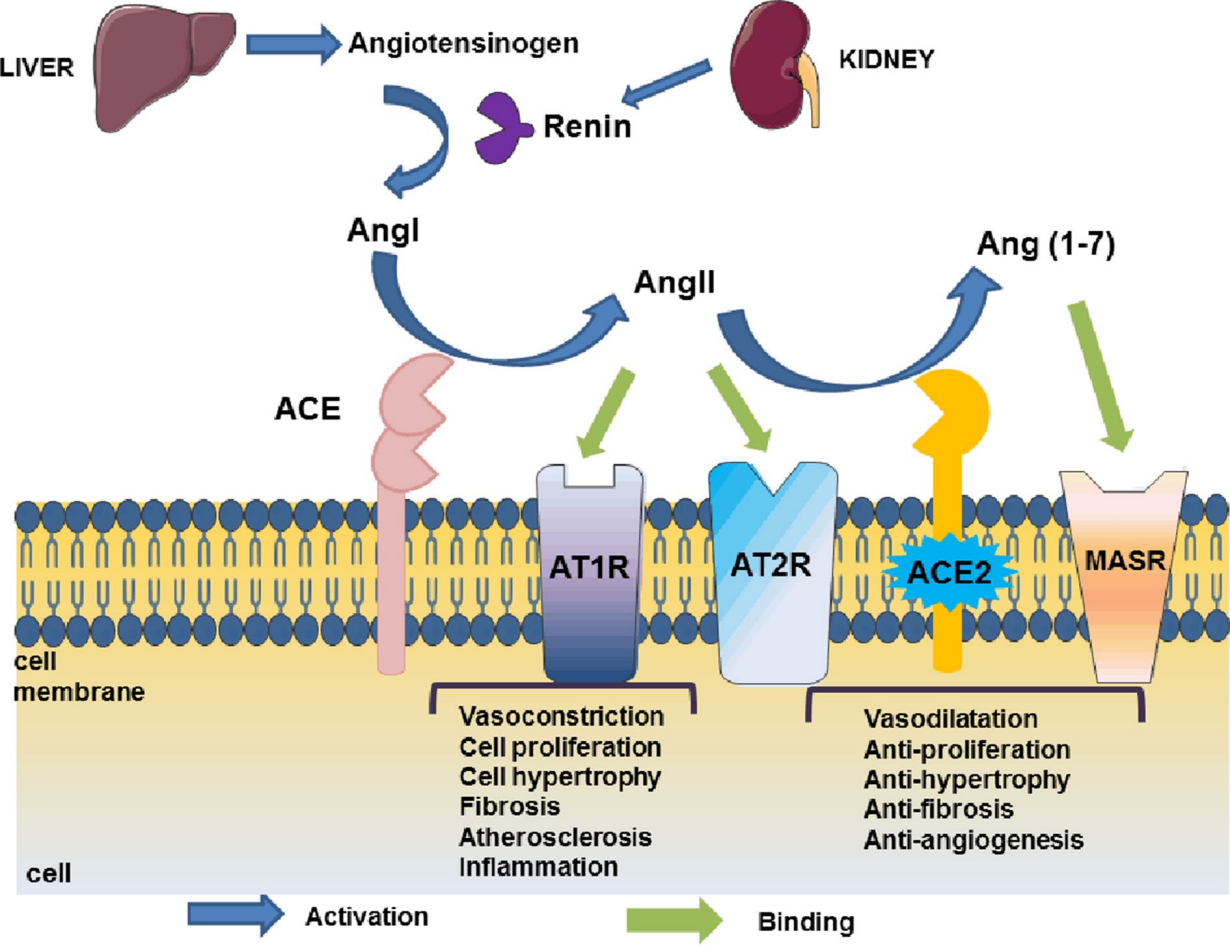
Schematic overview of RAS and its biologic functions. Angiotensinogen is secreted by the liver and is converted to angiotensin I (AngI) via renin, a protease produced in the kidneys. AngI is subsequently converted to AngII by the catalytic action of angiotensin-converting enzyme (ACE), and binds to Angiotensin II Type 1 (AT1) and Type 2 (AT2) receptors. AngII binds to the angiotensin type 1 receptor (AT1R) to promote actions, such as vasoconstriction, cell hypertrophy, fibrosis, proliferation and inflammation. ACE2 converts Ang-I and Ang-II to angiotensin (1–7). Ang (1–7) binds to the MAS receptor (MASR) to promote actions of vasodilation, vascular protection, anti-fibrosis, anti-proliferation, anti-inflammation and anti-angiogenesis. This figure was created using Servier Medical Art templates, which are licensed under a Creative Commons Attribution 3.0 Unported License; https://smart.servier.com

ACE2 is a key element of the RAS protective axis. In the first RAS enzymatic reaction, angiotensinogen is converted into Angiotensin I (Ang I) through the action of renin, an aspartyl protease. The generation of angiotensin II (AngII) by the action of angiotensin-converting enzyme (ACE), the main effector of the system, induces an increased blood pressure promoting vasoconstriction and inflammation (Fig. 2). Finally, ACE2 converts AngII to Ang-(1–7), a vasodilatory agent. The increase in the activity of ACE2 might attenuate the RAS system by inactivating and enhancing the production of Ang-(1–7). Ang-(1–7) acts mainly through the G protein-coupled receptor Mas. Ang-(1–7)/Mas axis and AngII/ATR2 are antagonists of the ACE/AngII/ATR1 receptor axis, especially under pathological conditions. The alternative ACE2/Ang-(1–7) axis of the RAS represents an endogenous counter-regulatory axis [23].

ACE2 expression is high on the luminal surface of intestinal epithelial cells, in this context, ACE2 functions as a co-receptor for nutrient uptake [44]. ACE2 has other essential actions such as a non-catalytic function and the regulation of renal amino acid transport and pancreatic insulin secretion [45].

## Role of ACE2 in pathogenesis of COVID-19

SARS-CoV-2 has a strong human cell-binding affinity to the ACE2 receptor and establishes the link between COVID-19 and the RAS [46]. According to Wu et al. [47] the only difference between SARS-CoV-2 and SARS-CoV is 380 amino acid substitutions. Furthermore, 27 amino acid substitutions were found in S protein with a length of 1273 amino acids, including six substitutions in the receptor-binding domain (RBD). The S protein contains a 3-D structure in the region that maintains the van der Waals forces and RBD contains a receptor-binding determining region (RBDR) that recognizes ACE2 [48]. The 394-glutamine residue in the RBD region of SARS-CoV-2 is recognized by the critical lysine 31 residue on the human ACE2 receptor [7].

**Fig. 3:**
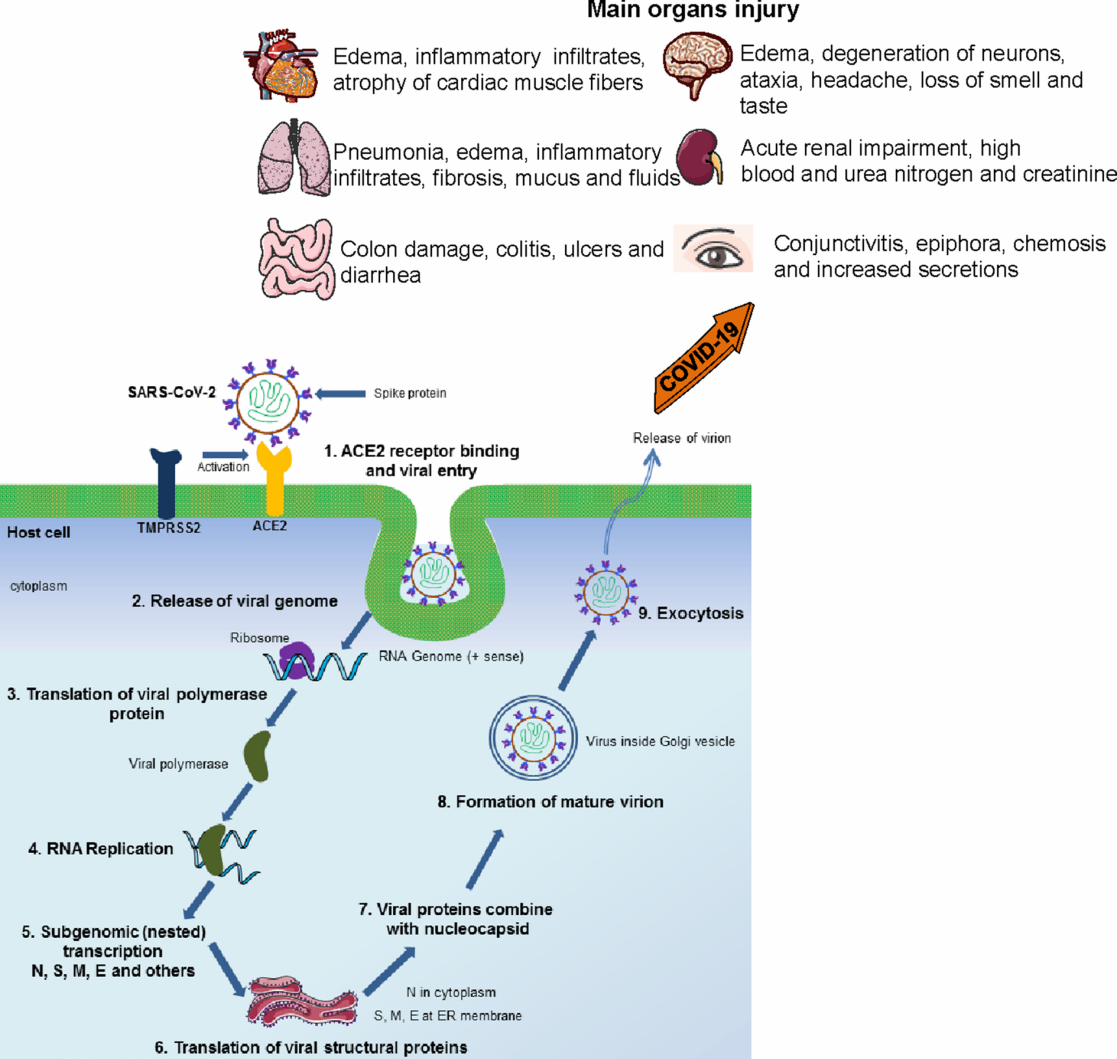
A simplified scheme of the life cycle of SARS-CoV-2 inside the host cell (with organ injury in COVID-19). (1) SARS-CoV-2 requires activation by the serine protease TMPRSS2 for optimal cell entry and the viral Spike glycoprotein of the virion binds to the cellular receptor ACE2 and enters target cells through an endosomal pathway. (2) Following the entry of the virus into the host cell, the viral RNA is released into cytoplasm. (3) After release of the viral genome the viral polymerase protein is translated from the genomic RNA. (4) Replication occurs and new ssRNA(+) are synthesized. (5) In transcription, a nested set of sub-genomic RNAs (sgRNAs) is produced (6) Viral structural proteins (S, E, and M) are translated from the RNA inserted into the endoplasmic reticulum, N in citoplasm and move to the endoplasmic reticulum-Golgi intermediate compartment. (7) The viral proteins formed in ER migrate to the Golgi apparatus and are assembled with the nucleocapsid. (8) Formation of mature virion. Finally, (9) the virions are released via the constitutive exocytic pathway out of the cell. In the SARS-CoV-2 infection multi-organ are injured in COVID-19 patients. S, spike protein; E, envelope protein; M, membrane protein; N, nucleocapsid protein; ER, endoplasmic reticulum. This figure was created using Servier Medical Art templates, which are licensed under a Creative Commons Attribution 3.0 Unported License; https://smart.servier.com

The entry of SARS-CoV-2 is mediated by its viral spike S glycoprotein, which binds to the ACE2 receptor and following activation of the spike protein by transmembrane protease serine 2 (TMPRSS2), and the entry is through endocytosis or membrane fusion (Fig. 3). The S protein is located on the outer envelope of the virion and has two functional subunits, S1 and S2, the first binds the cellular receptor, whereas S2 contains domains required for the fusion between viral and cellular membranes [7, 49–52]. Infection with SARS-CoV2 occurs upon viral binding and membrane fusion followed by internalization of ACE2 and down-regulation of its activity on the target cell surface [6]. The pathogenesis of COVID-19 is highly complex and the molecular mechanisms by which SARS-CoV-2 causes organ damage is yet unknown. The evidence clinical and progression of COVID-19 disease is consistent with direct viral effects and inflammatory and immune factors [53].

Studies have shown that SARS-CoV infection can downregulate ACE2 expression on cells and in pathological states resulting in elevated soluble ACE2 levels in blood, urine, and other body fluid [54]. In addition, can induces the downregulation of ACE2 and imbalance between the RAS and ACE2/angiotensin-(1–7)/MAS receptor axis and may also contribute to the multiple organ injuries in COVID-19 [54, 55]. Other studies have demonstrated that Ang II/AT1R axis can promote the COVID-19 progression causing vasoconstriction, inflammation, and fibrosis, and possibly leading to severe organ injury [56].

Furthermore, observations have suggested that ACE2 is upregulated by some ACE inhibitors (ACEIs) and possible increases in the expression of ACE2 induced by RAS inhibitors would have beneficial effects of protection against lung injury and other organ damage but not infection with SARS-CoV-2 [54]. The new disease, COVID-19 has potentiated the role of ACE2 as a receptor for SARS-CoV-2, but is necessary more research to understand whether ACE2 levels contribute to the COVID-19 pathogenesis and may explain the severe damage or could benefit the course of this disease by its downregulation.

## New mediator associated with SARS-CoV-2 viral entry: neuropilin-1

It is now well established that SARS-CoV-2 uses the receptor ACE2 to infect the cells, but viruses often use multiple factors to maximize their infectious potential. In addition to ACE2 and TMPRSS2, other potential SARS-CoV-2 receptors, proteases and cofactors for infection have been suggested, including BSG (CD147) and neuropilin-1 (NRP1). The C-terminal sequence may allow the protein to associate with cell surface NRP1 and NRP2 receptors. A recent finding suggests that NRP1 may serve as a host factor for SARS-CoV-2 infection, which is very abundant in many human tissues including the respiratory tract, blood vessels, and neurons [57]. NRP1 significantly potentiates SARS-CoV-2 infectivity. Daly et al. [57] showed that blocking the binding between NRP1 and C-end rule (CendR) motif in S1 (with RNAi or selective inhibitors), play a role in the increased infectivity of SARS-CoV-2 compared with SARS-CoV. They observed in cells with both the ACE2 and neuropilin-1 proteins, SARS-CoV-2 infection was greater compared to cells with either “doorway” alone.

NRP1 has been observed to be expressed in lung cells and in the olfactory epithelium more abundantly than ACE2 [58]. Thus, NRP1 may explain SARS-CoV-2 infection of alveolar cells despite the low expression of ACE2. Further characterization of NRP1 expression in the lung of healthy individuals and in patients with COVID-19 is needed to better understand its role in pathogenesis. Additional entry factors have been suggested, such as CD209L, CD209 and CD147/Basigin [59].

## ACE2 associations with several diseases and COVID-19

**Fig. 4:**
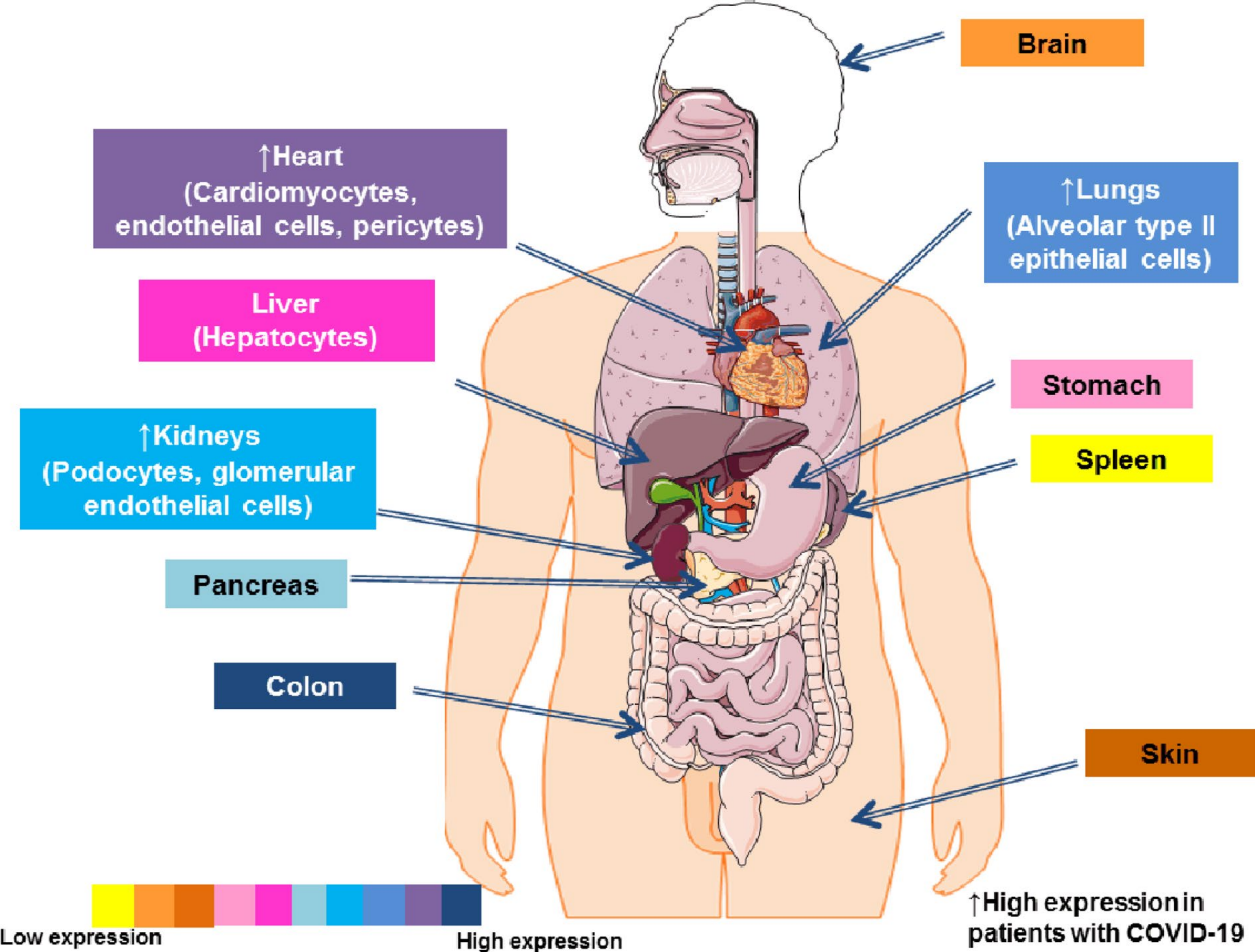
ACE2 expression in different organs in healthy individuals and individuals with COVID-19. The figure represents ACE2 expression in normal organs and cells, such as cardiomyocytes, podocytes and hepatocytes, which are colored by its level of expression. The expression of ACE2 is gradually increased from healthy spleen to the highest expression in colon. The up-arrow indicates high expression in kidneys, lungs and heart of patients with COVID-19. This figure was created using Servier Medical Art templates, which are licensed under a Creative Commons Attribution 3.0 Unported License; https://smart.servier.com

ACE2 is expressed in various organ systems, including the cardiovascular system, kidneys, brain and lung tissue, principally in Type II alveolar cells [60] (Fig. 4), and is associated with immunity, inflammation, increased coagulopathy, and cardiovascular disease.

In severe COVID-19 patients that present comorbidities, such as hypertension, diabetes, and chronic obstructive lung disease, ACE2 is highly expressed in the lungs [50]. The degree of severity may also be associated to the imbalance of ACE2 and the cytokine storm that results in heart failure progression [51] [preprint: not peer-reviewed]. Leung et al. [61] found increased ACE2 expression in lower airways of smokers and individuals with chronic obstructive pulmonary disease, but further investigation is required to verify the association between higher lung ACE2 expression and COVID‐19 susceptibility [62].

### SARS-CoV-2, ACE2 and diabetes

In a systematic review of 27 studies and 76,639 patients included, a 14.5% prevalence of diabetes in patients with COVID-19 was found. They also found that prognosis, severe symptoms, and the death rate is higher among patients with diabetes infected with SARS-CoV-2 [63].

Alterations of RAS have been associated to complications of diabetes mellitus (DM), including insulin resistance, endothelial damage and diabetic nephropathy due to elevated concentrations of AngII that contribute to increased oxidative stress and inflammation [52, 64].

**Fig. 5:**
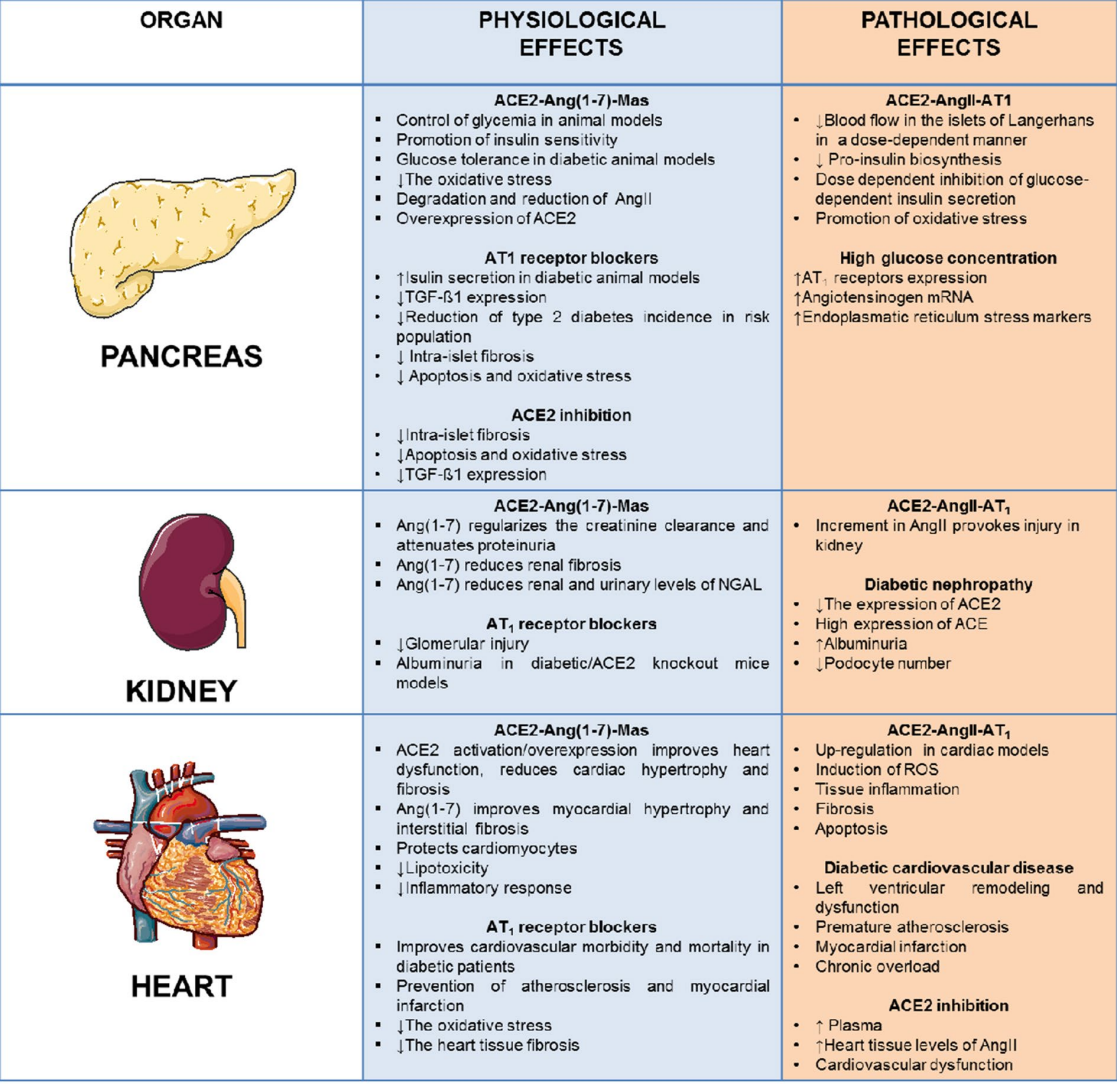
Physiological and pathological of RAS axes and ACE2 in different organs. The figure summarizes the role of components of the RAS system axes in the control of the glycemia, insulin secretion, diabetic nephropathy and in cardiovascular disease in diabetes. This figure was created using Servier Medical Art templates, which are licensed under a Creative Commons Attribution 3.0 Unported License; https://smart.servier.com

The role of the ACE2-Ang-(1–7)-Mas receptor axis is the focus of attention in the progression of diabetes mellitus and its complications, including poor glycemic control, diabetic nephropathy, kidney disease and cardiovascular alterations. ACE2 may act as a negative regulator of the classical RAS with a renoprotective effect and Fig. 5 summarizes the mains effects of the classical and the counter-regulatory RAS axes in the pancreas, kidney and heart [65].

The severity of COVID-19 in patients with diabetes, hypertension or other chronic diseases, may respond to activation of the RAS system in different tissues, leading to a compromised innate immunity, an inappropriate and elevated proinflammatory cytokine response and a low expression of ACE2. Diabetic patients with poorly controlled glycaemia have an increased risk of viral infections due to an altered immune response, caused by the impaired lymphocyte, neutrophil and monocyte/macrophage function, which also increases the speed of progression to septic shock and multiple organ failure [66–68].

The difference between diabetic patients and non-diabetic patients is the increase in the levels of pro-inflammatory markers, such as the leukocyte and neutrophil count, pro-calcitonin, C-reactive protein, ferritin, and circulating cytokines that trigger the cytokine storm, namely IL-6, IL-8, IL-2 receptor, TNF-α. This suggests that people with diabetes are more susceptible to an inflammatory cytokine storm eventually leading to acute respiratory distress syndrome (ARDS), shock and rapid deterioration of COVID-19 [67]. The potential pathogenic mechanisms that may increase the susceptibility for COVID-19 in patients with DM include dysfunctional glucose homeostasis, inflammation and insulin resistance, vascular endothelial damage, dysregulated immunological status and activation of the RAS system [69, 70].

Diabetes is another disease that has been studied widely to be associated to variants in the *ACE2* gene, there were 9 variants that were statistically significantly associated with *ACE2*. Some of the studies focused on assessing the association of the *ACE2* polymorphism and cardiovascular disorders in patients with Type 2 diabetes. Studies showed evidence that type 2 diabetes with certain ACE2 variations had a higher risk of cardiovascular complications such as coronary artery disease [71], hypertension, reduced systolic function, increased left ventricular mass [72, 73], dyslipidemia, carotid atherosclerosis stenosis, retinopathy [74].

### Hypertension and ACE2

Differences in blood pressure responses to AngII between males and females may result from estrogen-mediated increases in ACE2 and increased production of the vasodilator, Ang-(1–7) [37]. Hypertension is the disease that has been studied the most in relation to ACE2. There were 4 meta-analyses [75–77] published assessing the association between the ACE2 gene and essential hypertension. It was observed that the A allele of the G8790A polymorphism and the T allele of the rs2106809 polymorphisms were associated with essential hypertension risk [78].

### Cardiovascular disease and ACE2

In the last 15 years, elevation of serum ACE2 activity has been detected in several cardiovascular diseases, including heart failure, atrial fibrillation, aortic stenosis, coronary artery disease and myocardial infarction. It has also been associated to adverse outcomes and cardiac fibrosis [79–84]**.** ACE2 mRNA expression and ACE2 protein has been described in human atrial tissue [37, 82]. A recent study from Chen et al. [85], which created a cell atlas of the adult human heart with transcriptome analysis, observed that pericytes (cells intimately associated to endothelium in capillaries) were the cells with the higher expression of ACE2, suggesting a target for a potential SARS-CoV-2 infection in the heart. This work also observed increased ACE2 gene and protein expression in myocardial tissue from patients with heart failure, further confirming ACE2 as marker of cardiovascular disease.

A Chinese epidemiological study including more than 40 000 confirmed COVID-19 cases showed an overall case fatality rate of 2.3%, while the specific case fatality rate for patients with cardiovascular disease was 10.5%, implying enhanced susceptibility to death [86]. In contrast, there is no evidence of higher risk of SARS-CoV-2 infections for individuals with heart disease. New-onset heart failure [87] as well as myocardial injury determined by troponin elevation [88] have been reported in patients with COVID-19. However, it is unclear whether myocardial complications in these patients are the result of direct infection with SARS-CoV-2 or a secondary complication of inflammation and ARDS.

Gender-dependent differences in cardiac ACE expression with the enhanced cardiac ACE/RAS axis were compared in male vs. female mice. In females, the balance was shifted towards the alternative ACE2/Ang(1–7)/MasR and AT(2)R pathways. This could partially explain the obvious gender-specific differences in the prevalence of cardiovascular pathologies like myocardial hypertrophy, and cardiac fibrosis [30]. With aging, this cardiovascular protection in women is lost and this may be related to the loss of ACE2 in postmenopause and the possible shift from the protecting ACE2/RAS axis into the classical ACE/RAS pathways.

### ACE2, COVID-19 and coagulopathy

Several reports concerning disturbances in coagulation in COVID-19 patients have been published. Deep vein thrombosis was found in 7 of 12 individuals undergoing post-mortem assessment and 4 patients had pulmonary embolism as the direct cause of death [89]. In a multicenter study including 150 patients in an intensive care unit, 64 thrombotic complications were reported [90]. Also, patients with COVID-19, receiving anticoagulation therapy and with ARDS had significantly more thrombotic complications when compared with similar patients in the intensive care unit without COVID-19. Evidence of increased arterial thrombotic events has been published. In New York City, 5 patients younger than 50 years with COVID-19 were diagnosed with stroke, an increase in incidence of this disease compared to similar time frames in previous years [91]. The coagulation disorders observed in patients with COVID-19 may partially resemble the disseminated intravascular coagulation common in other patients with sepsis, however, it is more compatible with a distinctive hypercoagulable state [92].

Factors leading to thrombosis include endothelial damage, impaired blood flow or statis and hypercoagulability [93]. ACE2 expression in endothelial cells of capillaries from several human tissues was previously detected [49]. ACE2 mRNA expression was documented in pericytes, closely related to capillaries, and endothelial cells in tissue from human hearts [85], and ACE2-positive endothelial cells were detected in lung tissue from patients who died from COVID-19 [94]. These studies provided evidence indicating endothelial cells as possible entry sites for SARS-CoV-2. In a case series reporting the post-mortem examination of 3 patients that died due to COVID-19, endotheliitis was documented and imaging with electron microscopy showed the inclusion of viral particles in endothelial cells [95]. Injury to endothelial cells from infection may lead to endothelial activation. In fact, Von-Willebrand factor, which is released from endothelial cells upon activation, has been reported elevated in COVID-19 hospitalized patients compared with healthy controls [96]. This observation, in conjunction with other mechanisms such as hyper-viscosity, and complement activation might explain the coagulopathy present in COVID-19 [97].

Hypertension, diabetes and obesity are often associated with ACE2/Angiotensin 2 deregulation. ACE2, antagonizes angiotensin II, when the cell is infected by SARS-CoV-2, angiotensin II promotes vasoconstriction and vascular inflammation, associated with vascular thrombosis in arteries, veins, and capillaries and blood vessels. Angiotensin II-induced hypertension is accompanied by increased thrombosis. Clinical trials are now underway to address the vascular component of COVID-19. These include anticoagulant drugs to prevent thrombotic and thromboembolic disease, and RAS inhibitors [98].

### ACE2 and cancer

Expression of the RAS has been described in multiple tissues, such as liver, kidneys, or pancreas, as well as in cancer tissues, such as breast, colorectal cancer, and renal cell carcinoma. The expression of RAS components has been linked to the hallmarks of cancer due to an upregulation of such components in some cancer types [12, 13, 99]. The function of RAS has been involved in proliferative signaling, growth suppressor evasions, apoptosis resistance, angiogenesis, cellular energetics deregulation, inflammation, cellular migration, invasion and metastasis [100]. Cancer patients suffering novel coronavirus pneumonia face the risk of poor prognosis after infection due to the malignant tumor itself, chemotherapy and surgery [101].

ACE2 RNA is highly expressed in renal cancer and colorectal cancer, which was consistent with its expression in normal tissues [102, 103]. In certain types of cancer, the expression of ACE2 is significantly different in comparison to normal tissue, those include kidney chromophobe, breast invasive carcinoma, prostate adenocarcinoma, thyroid carcinoma, liver hepatocellular carcinoma and stomach adenocarcinoma [104]. A decreased ACE2 expression has been significantly associated with poor prognosis in overall survival of patients with kidney renal clear cell carcinoma mesothelioma, ovarian serous cystadenocarcinoma and liver hepatocellular carcinoma [104]. The association of the imbalance of the RAS pathway and cancer development in COVID-19 patients is something that needs to be studied in the near future.

### ACE2 and obesity

Obesity is one of the main risk factors for the severe form of COVID-19 [105]. There is evidence that the RAS system is closely related to obesity, energy metabolism, and food intake [106] due to an imbalance in the RAS system resulting in an overexpression of the ANGII and AT1R axis at the systemic levels [107]. ACE2 is largely expressed in adipose tissue and in greater quantities in visceral tissue [108]. Weight loss reverses the imbalance of the RAS in adipose tissue as well as at systemic levels [107].

### Clinical manifestations of COVID-19 and ACE2

Chen et al. [109] studied the immunohistochemistry in olfactory mucosa from individuals with and without chronic rhinosinusitis and found the presence of ACE2 in sustentacular cells. An independent study by Brann et al. [110] through single-cell RNA sequencing and immunohistochemistry confirmed the expression of ACE2 in sustentacular cells. Also, horizontal basal cells, a special population of cells responsible of renewing olfactory receptor neurons (ORN) after injury also expressed ACE2. Although ACE2 has not been reported in ORN, simultaneous damage to sustentacular cells and horizontal basal cells upon infection with SARS-CoV-2 could precede disturbances in olfaction, which have been reported in up to 98% of patients with confirmed COVID-19 [111].

Alterations in taste perception (independent of olfactory dysfunction) have been reported in individuals with COVID-19 [112]. Data from oral cavity available in the repository from The Cancer Genome Atlas (TCGA) unveiled a higher expression of ACE2 in oral tongue than in other oral cavity tissues [113]. RNA expression profile on tissue from the taste system has not been analyzed in humans, but infection of cells in taste buds remains a possibility. Interestingly, taste disturbances have been reported in healthy individuals after consumption of ACE inhibitors, suggesting a physiological involvement of the RAS in taste perception [114].

## ACE2 and the immune system

With the COVID-19 pandemic, the SARS-CoV-2 virus has increased the scientific interest and research efforts in targeting the coronavirus interaction with the host immune system. A robust inflammatory component associated to COVID-19 has recently led to an outstanding amount of investigations related to chemokine and cytokine production upon viral infection as well as to detailed characterizations of the innate and adaptive immune responses. To understand the immunopathogenesis of COVID-19, it is crucial to identify not only the molecular mechanisms mediating viral entry, propagation and consecutive tissue and organ damage, but also the interaction of the immune components with the virus.

Single cell RNA sequencing (scRNA-seq) studies provide signals at a molecular level and at single-cell resolution that are complementary to bulk cell phenotypical and functional characterizations by techniques such as flow cytometry or mass spectrometry. Access to scRNA-seq data banks has contributed to expand and to complement studies based on independent observations. Integrated analysis of scRNA-seq to study associations of the receptor for SARC-CoV-2 (ACE2) and its mediators (TMPRSS2, CTSB and CTSL) for viral entry have provided new data on multiple gene networks involved in the inflammatory process occurring in COVID-19 [115].

To describe the infection process of SARS-CoV-2 more in detail, one of the largest studies integrating independent single-cell and single-nucleus RNA-seq data, analyzed data from 107 different studies assessing cell type-specific RNA expression of ACE2, TMPRSS2 and CTSL on lung and airways as well as other diverse organs. A striking finding related to cells of the immune system is the high expression of TMPRSS2 and ACE2 in NK and T cells in comparison to their immune cells [38] [preprint: not peer-reviewed]. Whether this has an impact on the innate and adaptive-mediated immune responses either by cell activation, cell depletion or other mechanisms, remains open for further investigation. Multiple shared gene programs on ACE2 regulation related to immune responses in nasal, lung and gut tissues have been identified in dual positive ACE2 + TMPRSS2 + cells when compared to dual negative cells. Such gene expression programs include IL-6, IL1R and TNF signaling, as well as immune functions in cross-talk between AT2 cells and macrophages [38] [preprint: not peer-reviewed].

The main immune pathophysiological events that gradually lead to a severe presentation of COVID-19 include a systemic inflammatory response syndrome and a dysregulated immune response [113]. Such events respond to a hyperactivation of the innate immune system caused by inhibition of interferon signaling by the virus and the production of proinflammatory cytokines, in particular IL-6 and TNFa. In line with this, studies using airway epithelial cells have revealed genes important for the development of an immune response against viral infection (i.e. IDO1, IRAK3, NOS2, TNFSF10, OAS1, MX1) suggesting the role of interferons in ACE2 regulation [33].

Lymphopenia is considered an important predictor of prognosis for disease severity in COVID-19 [116, 117]. Possible mechanisms of reduced blood lymphocytic levels include the direct or indirect effect of viral entry to immune cells. In the first case, since lymphocytes express ACE2 [38], a viral infection might result in selective lymphocyte death, whereas through an indirect mechanism, the virus might damage lymphatic organs such as the spleen and thymus leading to an acute decline of white blood cells. Another possible mechanism could be apoptosis-mediated lymphocyte depletion, as observed in infections by other coronavirus [118].

The high expression of ACE2 in endothelial cells and the exposition to SARS-CoV-2 may generate an environment of constant inflammatory changes reflecting other clinical markers indicative of immune response such as leukocytosis (mainly neutrophilia), thrombocytopenia [119], endothelial damage and activation leading to thrombosis and critical illness [120] as seen in other viral infections [121, 122] but at a higher frequency in COVID-19 [120, 123].

## ACE2 and treatment for COVID-19

The ACE2 is as possible target for preventing SARS-CoV-2 entering into the cell, therefore the ACE2 and the TMPRSS2 have been key to identifying candidate molecules/drugs that can stop SARS-CoV-2 of penetrating the human cell. Few drug candidates may inhibit infection and replication of SARS-CoV-2 such as inhibitors of TMPRSS2 serine protease and inhibitors of ACE2. Hoffman et al. [124] recently demonstrated that 2019-nCoV-S uses the SARS29 coronavirus receptor, ACE2, for entry, whereas the cellular protease TMPRSS2 for 2019-nCoV30 S for priming, showing that the inhibition of TMPRSS2 in human lung Calu-3 cells by camostat mesilate significantly reduced infection with SARS-CoV-2. Camostat mesylate, a serine protease inhibitor approved in Japan to treat unrelated diseases, has been shown to partially block and significantly reduce infection by SARS-CoV and HCoV-NL63 in HeLa cell expressing ACE2 and TMPRSS2 [125]. Another protease inhibitor with a broad-spectrum serine protease inhibitor function, nafamostat mesylate (NM) blocks SARS-CoV-2 infection of human lung cells with a markedly higher efficiency than camostat mesylate [126]. NM is approved in Japan and South Korea for the treatment of pancreatitis, disseminated intravascular coagulation, and systemic inflammatory response syndrome via suppression of thrombin, plasmin, kallikrein, trypsin, and Cl esterase in the complement system, as well as factors VIIa, Xa, and XIIa in the coagulation cascade [127–130].

A clinical-grade soluble recombinant human ACE2 protein (hrsACE2) inhibits the attachment of SARS-CoV-2 to simian Vero-E 6 cells and inhibits infection, but the inhibition was not complete and was dose-dependent [29]. A recently reported case of a 45-year-old woman with severe COVID-19 that did not respond to treatment, showed that after receiving hrsACE2, the viral load was rapidly reduced in serum along with the generation of anti-SARS-CoV-2 IgA and IgG antibodies [26]. Thus, targeting the soluble form of ACE2 could have an important effect on blocking the systemic spread of the virus from the lung to other organs. Researchers are studying other promising drugs for the treatment of the SARS-CoV-2 infection relying on the molecular docking results obtained in comparison with the ligand N3. One study analyzed the drugs aliskiren, dipyridamole, mopidamol, rolitetracycline, metamizole, and rosuvastatin. The author found that aliskiren had the highest score of binding with the binding site of N3, the advantage of renin inhibition, and the possibility of the reduced expression of ACE2 [131] [preprint: not peer-reviewed]. Other therapies under study include the use of monoclonal antibodies against COVID-19 (e.g. B38, H4, 47D11) targeting the receptor-binding domain (RBD) to inhibit the union of the virus with the ACE2 receptor [132] which has shown promising results for the treatment and/or prevention of COVID-19.

Fan et al. [133] screened two libraries of 2406 clinically approved drugs to study the ability to inhibit cytopathic effects on Vero E6 cells by GX_P2V/pangolin/2017/Guangxi infection and found that only the combination of cepharanthine, selamectin, and mefloquine hydrochloride was identified as a candidate drug combination against SARS-CoV-2 infection.

One of the first candidate drugs were Chloroquine (CQ) and hydroxychloroquine (HCQ) because they inhibit the terminal phosphorylation of ACE2 and elevate the pH in endosomes, respectively [134]. However the RECOVERY and the SOLIDARITY trials have shown a lack of efficacy and serious adverse events [135, 136].

Professional societies [137, 138] as well as the EMA, do not support the discontinuation of ACEI/ARB. Studies like the one from Fosbøl et al. [139] recently demonstrated that prior use of ACEI/ARBs, among patients diagnosed with COVID-19, was not significantly associated with COVID-19 diagnosis among patients with hypertension or with mortality or severe disease. Another observational study of 1,591 patients demonstrated that antihypertensive therapy did not significantly interfere with COVID-19 lethality. More randomized studies like the BRACE CORONA trial (NCT04364893) are needed to evaluate whether to continue or suspend treatment.

There has been some controversy about the use of ACE inhibitors and angiotensin-receptor blockers (ARBs) in COVID-19 patients or if this could be associated with the risk of infection by increasing the ACE2 expression. However, other studies have shown that treatment with ARBs may mitigate angiotensin II-mediated lung injury by blocking the AT1 receptors. More studies are needed to elicited the use of RAS inhibitors, especially in patients with preexisting conditions [98].

Ghazizadeh et al. [140] [preprint: not peer-reviewed], have demonstrated an explanation about two important observations in the COVID-19 pandemic: the higher prevalence of severe complications in male individuals and the relative immunity in children and is due to a link between male sex hormone signaling and regulation of the SARS-CoV-2 receptor ACE2 and co-receptor TMPRSS2. Their results demonstrated that inhibitors of 5 alpha reductases can reduce ACE2 levels and decrease internalization of the viral spike-RBD.

ACE2 is one of the principal candidates for drug development to treat COVID-19. There are proposals to engineer human ACE2 to optimize the binding to the spike protein of COVID-19, and several monoclonal antibodies with exceptional affinity for protein S are being developed [141].

Given that COVID-19 have different stages (severities), it will be necessary to study these drugs according to the different stages of the disease. In addition, given that men have a higher risk of severe COVID-19 and the differences observed in the gene, studies should stratify by sex.

## CONCLUSION

ACE2 is the doorway through which SARS-CoV-2 gains entrance into human cells, with the spike proteins of the virus being the keys that unlock the doorway. The spike proteins have high affinity to ACE2. These spikes bind to the ACE2 membrane, freeing the virus to enter the human cell. These binding mechanisms have been proposed to be essential in the understanding of the physiopathology of severe forms of COVID-19. For the prevention, treatment as well as the development of drugs and vaccines, it is imperative to understanding the RAS system and particularly the role that ACE2 plays in the pathophysiology to identify patients that are susceptible to severe forms of COVID-19.

This review provides an overview of the different components of ACE2, starting from the gene, its protein and its expression to provide the different pieces of the puzzle. In addition, it reviews the different comorbidities (eg. diabetes, hypertension, obesity) that interact with SARS-CoV-2 in which also ACE2 plays an important role. It also described the different factors that interact with the virus that have an influence in the expression and functional activities of the receptor. The goal is to provide the reader with an understanding of the complexity and importance of this receptor.

## Supplementary Information

**Additional file 1.** Genetic variants of *ACE2* associated with diseases.

## Data Availability

Not applicable.
