# Supplementary material for: ACE2: the molecular doorway to SARS-CoV-2
Source: Cell Biosci. 2020 Dec 30;10:148. doi: 10.1186/s13578-020-00519-8 (PMC7772801; doi:10.1186/s13578-020-00519-8)
Supplement: Supplementary file 1 — Additional file 1. Genetic variants of ACE2 associated with diseases. [file 13578_2020_519_MOESM1_ESM.docx]

**Table S1**

| **DISEASE** | **VARIANT** | **LOCALIZATION** | **ALT** | **FUNCTION** | **CODING** | **REFERENCE** |
| --- | --- | --- | --- | --- | --- | --- |
| Essential Hypertension | A | rs2285666 | splice_region_variant+intron_variant | NM_021804.2:c.439+4G>A | Coding variant | 25237167, |
|  | T | rs2106809 | intron_variant | NM_021804.2:c.186+788T>C | No | 22297693, 22988445, 19286756 |
| Atrial Fibrillation | T | rs2106809 | intron_variant | NM_021804.2:c.186+788T>C | No | 24342297 |
| Diabetes Mellitus, Type 2 | T | rs1978124 | intron_variant | NM_021804.2:c.186+786A>G | No | 30227878 |
|  | T | rs2048683 | intron_variant | NM_021804.2:c.584-920A>C | No | 20592051 |
|  | CC | rs2074192 | intron_variant | NM_021804.2:c.2115-449G>A | No | 20592051 |
|  | A | rs2106809 | intron_variant | NM_021804.2:c.186+788T>C | No | 20592051 |
|  | C | rs233575 | intron_variant | NM_021804.2:c.2115-625C>T | No | 20592051 |
|  | C | rs4240157 | intron_variant | NM_021804.2:c.1897-1015G>A | No | 20592051 |
|  | A | rs4646156 | intron_variant | NM_021804.2:c.1071-605T>A | No | 20592051 |
|  | T | rs4646188 | intron_variant | NM_021804.2:c.901-1830T>C | No | 20592051 |
|  | C | rs879922 | intron_variant | NM_021804.2:c.1542-361G>C | No | 20592051 |
| Dilated Cardiomyopathy | C | rs6632677 | intron_variant | NM_021804.2:c.187-1746C>G | No | 28744816 |
| High-density lipoprotein cholesterol (HDL-C) | A | rs2285666 | splice_region_variant+intron_variant | NM_021804.2:c.439+4G>A | Coding variant | 20813695 |
| Hypertrophic cardiomyopathy | T | rs2106809 | intron_variant | NM_021804.2:c.186+788T>C | No | 18560893 |
|  | C | rs6632677 | intron_variant | NM_021804.2:c.187-1746C>G | No | 18208662 |
| High sensitivity C-reactive protein (hsCRP) | A | rs2285666 | splice_region_variant+intron_variant | NM_021804.2:c.439+4G>A | Coding variant | 20813695 |
| Intima-media thickness (IMT) | A | rs2285666 | splice_region_variant+intron_variant | NM_021804.2:c.439+4G>A | Coding variant | 20813695 |
| Left Ventricular Hypertrophy | C | rs233575 | intron_variant | NM_021804.2:c.2115-625C>T | No | 16283142 |
|  | G | rs4240157 | intron_variant | NM_021804.2:c.1897-1015G>A | No | 16283142 |
|  | T | rs4646156 | intron_variant | NM_021804.2:c.1071-605T>A | No | 16283142 |
|  | G | rs879922 | intron_variant | NM_021804.2:c.1542-361G>C | No | 16283142 |
| Pulse pressure | A | rs2285666 | splice_region_variant+intron_variant | NM_021804.2:c.439+4G>A | Coding variant | 20813695 |
| Small for Gestational Age | T | rs2074192 | intron_variant | NM_021804.2:c.2115-449G>A | No | 30347406 |
